# Databases of homologous gene families for comparative genomics

**DOI:** 10.1186/1471-2105-10-S6-S3

**Published:** 2009-06-16

**Authors:** Simon Penel, Anne-Muriel Arigon, Jean-François Dufayard, Anne-Sophie Sertier, Vincent Daubin, Laurent Duret, Manolo Gouy, Guy Perrière

**Affiliations:** 1 Laboratoire de Biométrie et Biologie Évolutive, CNRS, Université Claude Bernard – Lyon 1, 43 bd. du 11 Novembre 1918, 69622 Villeurbanne Cedex, France; 2 Laboratoire d'Informatique, de Robotique et de Microélectronique de Montpellier, 161 rue Ada, 34392 Montpellier, France

## Abstract

**Background:**

Comparative genomics is a central step in many sequence analysis studies, from gene annotation and the identification of new functional regions in genomes, to the study of evolutionary processes at the molecular level (speciation, single gene or whole genome duplications, etc.) and phylogenetics. In that context, databases providing users high quality homologous families and sequence alignments as well as phylogenetic trees based on state of the art algorithms are becoming indispensable.

**Methods:**

We developed an automated procedure allowing massive all-against-all similarity searches, gene clustering, multiple alignments computation, and phylogenetic trees construction and reconciliation. The application of this procedure to a very large set of sequences is possible through parallel computing on a large computer cluster.

**Results:**

Three databases were developed using this procedure: HOVERGEN, HOGENOM and HOMOLENS. These databases share the same architecture but differ in their content. HOVERGEN contains sequences from vertebrates, HOGENOM is mainly devoted to completely sequenced microbial organisms, and HOMOLENS is devoted to metazoan genomes from Ensembl. Access to the databases is provided through Web query forms, a general retrieval system and a client-server graphical interface. The later can be used to perform tree-pattern based searches allowing, among other uses, to retrieve sets of orthologous genes. The three databases, as well as the software required to build and query them, can be used or downloaded from the PBIL (Pôle Bioinformatique Lyonnais) site at .

## Background

HOVERGEN, a database devoted to homologous gene families in vertebrates [1,2] has been first released in 1994. The motivation to develop this database was to build a system allowing to do large-scale comparative genomic studies on vertebrates. HOVERGEN allows to retrieve sets of orthologous genes in order to do evolutionary studies on gene families [3-12].

Two other systems based on the same architecture: HOGENOM and HOMOLENS are presented here. HOGENOM contains homologous gene families from all available complete genomes from bacteria, archaea and unicellular eukaryotes, plus some representative plants and animals. HOMOLENS contains gene families from complete animal genomes found in Ensembl [13]. In the three databases, after family assembly, protein sequences are aligned and the alignments produced are used to build phylogenetic trees. Those two steps are realized through an automated procedure.

These databases are structured under the ACNUC sequence database management system [14]. Access to these databases is possible through different implementations of the ACNUC libraries. The first one is the Web server available at PBIL [15]. The second one is the program Query, a retrieval system allowing to query local or remote ACNUC databases [16]. Lastly, a graphical interface named FamFetch allows to retrieve families and display associated data [17,18]. This program allows to perform pattern searches on the phylogenetic trees through a pattern-matching algorithm. This feature is especially helpful to retrieve sets of orthologous sequences, but also for any kind of studies involving the detection of phylogenetic profiles.

## Materials and methods

### Data harvesting and pre-processing

For the three systems, two ACNUC databases are built, one for the protein sequences and one for the corresponding nucleotide sequences. Protein sequences are stored in UniProtKB format [19] while nucleotide sequences are stored in EMBL format [20]. To build those databases, the sequences are gathered from different sources. In the case of HOVERGEN, protein sequences represent the primary source of information, and they are taken from UniProt. Nucleotide sequences are taken from EMBL, using the cross-references provided in UniProt. For HOMOLENS, nucleotide annotated sequences come from Ensembl and protein sequences are generated from the corresponding Coding DNA Sequences (CDS) described in Ensembl annotations. In the case of HOGENOM, data sources are represented by various nucleotide sequence collections that are used in a hierarchical manner. Sequences from Genome Reviews [21] are used first and then supplemented with various systems such as Ensembl, the NCBI microbial data repository and complete genomes collection, the European Bioinformatics Institute (EBI) complete genome data, sequences from the Joint Genome Institute (JGI), the Sanger Institute and the John Craig Venter Institute (JCVI). The CDS from these collections are translated, using the adequate genetic code and reading frame, to generate the corresponding protein sequences except when alternative splicing occurs. In this case only the longest CDS is translated. Annotations of the CDS are analysed to get information related to protein annotations. When cross-references to UniProt are found, UniProt entries are scanned to get information on function, product and bibliography to improve the annotations. The UniProt identifier is inserted into the annotations as a keyword and the UniProt accession number is inserted as a secondary accession number.

Inconsistencies or lack of precision in the taxonomic information present in some source databases are corrected, mostly in HOGENOM. In HOVERGEN, UniProt and EMBL sequence names and accession numbers are used. In HOGENOM and HOMOLENS, devoted to complete genomes, entries are renamed to directly provide information about the species identity and the location of genes in chromosomes. For nucleotide sequences, the first two letters of the genus, the first three letters of the species, a number identifying the strain, and another identifying the replicon, the chromosome, or the organelle make up sequence names. For each individual CDS, a suffix containing the two letters "PE" (for peptide) followed by its rank number in the replicon is added to the containing sequence's name. For example, ESCOL2_1.PE76 and ESCOL2_2.PE3371 correspond respectively to the sequence of the *traL *gene on plasmid *F *and the sequence of the *glgX *gene on the chromosome of *Escherichia coli *K12. For protein sequences, the same naming is employed, except that the CDS rank number is integrated in the sequence name (*e.g.*, ESCOL2_1_PE76 for the above mentioned *traL *gene). Note that original accession numbers are conserved and added to sequence annotations so that the coherence with original data source is conserved.

### Clustering algorithm

**Figure 1:**
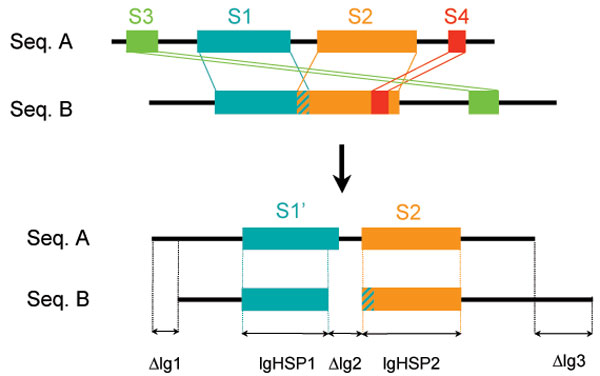
**Removal of incompatible HSPs**. For each couple of homologous sequences found by BLASTP, HSPs that are incompatible with a global alignment are removed. In this example, segments *S1 *and *S2 *are compatible, but segments *S3 *and *S4 *are not. They are therefore ignored by further computations on similarity measures which allow one to classify (or not) these two sequences in the same family.

To build families, a similarity search of all proteins against themselves, after filtering low complexity regions with SEG [22], is performed with the BLASTP2 program [23], the BLOSUM62 amino-acid similarity matrix [24], and a threshold of 10^-4 ^for BLAST *E*-values. The Build_Fam program is used to cluster protein sequences into families. This program filters BLAST output in order to remove Homologous Segment Pairs (HSPs) that are incompatible with a global alignment (Figure 1). For complete protein sequences, two sequences in a pair are included in the same family if remaining HSPs cover at least 80% of the protein length and if their similarity is over 50% (two amino-acids are considered similar if their BLOSUM62 similarity score is positive). This couple of parameters will be denoted by 50/80 below.

Build_Fam uses a simple transitive link to build families. It means that if the pair of sequences {A, B} matches the conditions to be integrated in the same family and if the pair {A, C} also matches them, then sequences A, B and C will be clustered together, even if the pair {B, C} does not match the conditions. Once families of complete protein sequences are built, partial sequences are included in the classification. A partial sequence having similarity with a complete protein is included in a family if it fulfils the two conditions required for a complete sequence and if its length is ≥100 amino-acids or ≥50% of the length of the complete protein. When several families can be associated with a partial sequence, the sequence is included in the family that presents the complete sequence with the highest similarity.

### Extensions of sequence annotations

Further sequence annotations are created after the clustering step. For protein sequences, a family identifier is added in the "CC" field. In the case of nucleotide sequences, this information is added in a "/gene_family" qualifier associated to each CDS. In both cases, this identifier is incorporated in the keywords associated to the corresponding entries in the ACNUC structure. It is thus possible to retrieve all the sequences in a family with this number when using any of the retrieval systems developed for our three databases. Some supplementary features corresponding to descriptions of non-coding regions are also introduced in the nucleotide sequences: "INT_INT" for internals introns (*i.e.*, within CDS), "5'NCR" for 5' non-coding regions, and "3'NCR" for 3' non-coding regions (*i.e.*, regions respectively upstream and downstream of annotated CDS, including UTRs and intergenic regions). Those supplementary features define what we call sub-sequences [14] which can be selected and extracted from the databases in the same way as CDS or structural RNAs.

### Alignments and phylogenetic trees

Once the families are built, multiple alignments are computed on protein sequences using MUSCLE [25] with all default parameters. Alignments are filtered with Gblocks [26] in order to keep only their reliable parts. Based on our experience, Gblocks is used with parameters corresponding to relaxed conditions, in agreement with Talavera and Castresana [27]. Phylogenetic trees are computed with the fast maximum-likelihood algorithm implemented in PhyML [28], the JTT amino acid substitution model [29], and across-site rate variation modelled by a gamma distribution with four rate classes. Estimation of the *α *parameter for gamma distributions is carried out by PhyML. Internal branch support is estimated using the approximate Likelihood Ratio Test (aLRT) available in PhyML [30]. Due to the amount of time and memory required by computations on large families, alignments and tree computations were limited to families up to 1,000 sequences in HOVERGEN and up to 2,000 sequences in HOGENOM and HOMOLENS.

### Tree reconciliation

**Figure 2:**
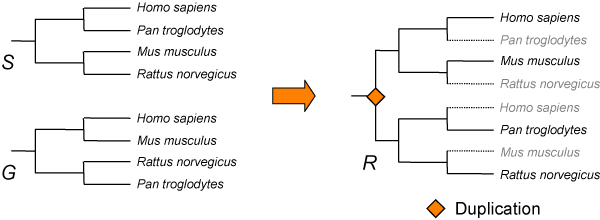
**Tree reconciliation between a gene tree *G *and a species tree *S *showing different topologies**. The result is the reconciled tree *R*. *R *is a variation of *S*, in which duplication nodes have been inserted in order to explain incongruence with *G*.

All individual phylogenetic trees are reconciled with a species tree using the program RAP [18]. The reconciliation consists in the comparison of a gene tree with a species tree. When inconsistencies are detected between the two, they are explained by the presence of duplication events followed by selective losses in different lineages (Figure 2). The reference species tree used is the one provided by the NCBI taxonomic database . During this process, some annotations are added to the reconciled trees. Those annotations consist in taxonomic data (*i.e.*, species names) and cross-references to the CDS corresponding to the protein sequences used to build the trees. Trees are rooted using the same reconciliation procedure. The root is placed to maximize the similarity between the gene tree and the species tree. All possible positions of the root in the gene tree are explored, and the one that requires the minimal number of gene duplications is retained. Tree reconciliation is used for HOVERGEN and HOMOLENS but not for HOGENOM because RAP does not model Horizontal Gene Transfers (HGTs), which are thought to be an important source of phylogenetic inconsistencies in prokaryotes [31-33].

### Evaluation of clustering criteria

The efficiency and reliability of our clustering algorithm was assessed through a comparison with alternative approaches. We selected all the 219,951 protein sequences from 50 complete genomes including a panel of bacterial, archaeal and eukaryotic species in HOGENOM. For Build_Fam, three similarity/length-percentage combinations were experimented: the above-mentioned 50/80 and also the 40/80 and 40/90 combinations. We also applied the OrthoMCL and TribeMCL clustering programs on the same dataset. OrthoMCL is used to build the OrthoMCL-DB database [34]. This approach attempts to use evolutionary concepts such as orthology (*i.e.*, divergence after speciation events) and paralogy (*i.e.*, divergence after duplication events) to enforce a lower weight to paralogous relationships during the MCL clustering procedure [35]. This algorithm uses an inflation parameter (*I*) which regulates the cluster tightness. The default value for OrthoMCL is *I *= 1.5, but we also examined its behaviour with *I *= 1.1 and 4.0. TribeMCL is the algorithm used to build Tribes [36], and it is based on a similarity criterion provided by the user. Two different similarity criteria for TribeMCL were used: i) the simple BLAST *E*-value; and ii) our own score, Tribe(HSP), defined as:

(1)

where *x *and *y *are two homologous protein sequences, *s*(*HSP*_*xy*_) is the BLAST bit score for an HSP in an ordered list of HSPs found between *x *and y, and *s*_*zz *_is the BLAST bit score between sequence *z *and itself. The value given to the inflation parameter for MCL in this case was the default one (*I *= 2).

The desired properties of a clustering algorithm for phylogenetic database reconstruction are twofold: first, the algorithm should be able to cluster homologous sequences from divergent organisms; second, the resulting alignments should nevertheless remain of high quality. After clustering, families based on each algorithm were aligned using MUSCLE with default parameters. To estimate the quality of alignments, six subsets of families were considered for each clustering algorithm: three containing all families with 10, 25 and 50 sequences, and three containing all families of 10, 25 and 50 species. The quality of alignments was assessed using two approaches: the NorMD index [37] which computes a similarity score over the entire alignment based on amino acid similarity (measured with PAM250 in this study); and Gblocks filtering [26] which we used as a measure of the number of gaps introduced in the alignment. When NorMD ≥ 0.5, the alignment is considered to be of good quality [37]. For Gblocks, the higher the percentage of sites conserved after filtering, the better the alignment. We used the default parameters for Gblocks (all gaps are removed), and we considered empirically that the alignments were of good quality if the percentage of conserved sites was ≥50%.

### Databases access

As of October 2008, HOGENOM and HOMOLENS gather the information of complete genomes from respectively 513 and 41 species, while HOVERGEN contains 415,383 vertebrate proteins, and these three databases are regularly updated. They all provide high quality alignments and phylogenetic trees that can be queried and downloaded using a wide variety of tools, allowing to perform from very simple text searches to complex queries. Contents in terms of sequences and families for the present releases of the three databases are given in Table 1.

**Table 1:** Databases content for HOVERGEN, HOGENOM and HOMOLENS.

	HOVERGEN	HOGENOM	HOMOLENS
Proteins	415,383	2,142,639	672,064
CDS	613,473	2,128,552	892,572
Genomic sequences	541,405	135,105	178,069
Families	16,673	147,586	23,155
Orphans	24,234 (5%)	397,545 (18%)	90,953 (13%)
Proteins associated to a family	311,647 (75%)	1,742,390 (81%)	579,620 (86%)
Unclassified partial sequences	79,502 (19%)	-	-

### Web services

Sequences and families can be selected and retrieved via the PBIL server . This server provides convenient and flexible web forms for selecting sequences and families by many different criteria in several databases [38], including the general repository collections such as Ensembl, UniProt, GenBank [39] or EMBL. The core of the service is represented by the WWW-Query application [15]. The corresponding form allows the combination of up to four criteria to retrieve sequences or gene families. Among the allowed criteria are: sequence names, accession numbers, keywords, taxonomic data, organelle, molecule type (CDS, RNA, or the supplementary features described in the **Extensions to sequence annotations **section), bibliographical references, date of insertion in the repository collections. Each time a query is performed, the list of matching sequences is stored on the server, and it is possible to re-use previously created lists to refine queries. The Quick Search form represents a simpler version of this application. With this form, the user enters only a string corresponding to a sequence name, an accession number, a keyword or a species name, and all the sequences or families associated to a criterion matching the string will be sorted. Note that the use of wildcard for fuzzy searches is allowed with both WWW-Query and Quick Search.

The Cross Taxa application gives access to a family retrieval system based on taxonomic criteria. It allows to retrieve gene families that are shared by a first set of taxa and (optionally) that are not present in a second set of taxa. Any taxonomic level can be used and mixed to compose the query (*e.g.*, *Homo sapiens*, Mammalia, Metazoa). For example it is possible to retrieve all gene families specific to a toxic bacterial strain, all gene families present in human but not in rodents, or all metazoan-specific gene families.

**Figure 3:**
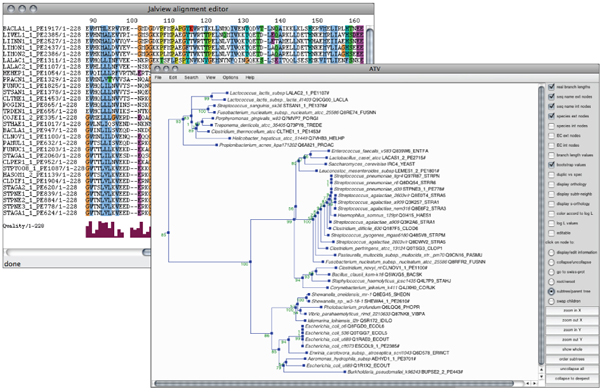
**Multiple alignments and phylogenetic trees visualization through the PBIL Web interface**. In this exemple, the alignment is displayed with the JalView applet and the phylogenetic tree is displayed with the ATV applet.

Alignments can be displayed on static HTML pages with several colouring options and they can be edited in order to visualize only a subset of sequences (Figure 3). Alternatively they can be visualised with the JalView applet [40] or downloaded on local disk. Phylogenetic trees are displayed as a clickable Portable Network Graphics (PNG) picture generated with Perl modules [41] and coloured according to taxonomy. Several displaying options are available, allowing to visualize species names, sequence name. Alternatively, trees can be visualised with the ATV applet [42] or downloaded.

Standard BLAST similarity searches can be performed on the three databases, but it is also possible to use a specific tool named HoSeqI [43]. With HoSeqI, instead of simply identifying the sequences in a database that are the most similar to a query sequence, the application identifies the most similar family. Then the query sequence is integrated into this family and the corresponding alignment and tree are recomputed on the fly. For that purpose, a panel of different multiple alignment and tree building programs is proposed to the user. Especially, it is possible to use profile alignments algorithms instead of performing *de novo *complete alignments. Therefore, the complete identification process can be very fast. Again, alignments and trees can be visualized on static HTML pages or through the use of JalView and ATV applets.

Lastly, note that HOVERGEN and HOGENOM families and phylogenetic trees can be directly accessed from the UniProt Web site , through cross-references of the "Phylogenomic databases" field.

### ACNUC remote connection

The ACNUC database system handles any sequence collection structured with the GenBank, EMBL or UniProt flat file formats. Recently, network access to ACNUC databases has been achieved by the definition and implementation of a remote ACNUC access protocol that governs information exchanges between the PBIL and remote clients [16]. This protocol uses a TCP/IP socket connection to a dedicated server and makes retrieval operations to remote ACNUC databases nearly as fast as to local databases with usual academic Internet connections.

HOVERGEN, HOGENOM and HOMOLENS can be accessed with two client programs: Query_win with a graphical user interface, and raa_query with a command-line interface. The latter is useful in a scripting context, possibly to repeatedly execute fixed retrieval operations. Both of them allow to compose complex queries involving multiple criteria, extraction of sequences and sub-sequences into local files, and access to keywords and taxonomic data browsers. Query_win executables are available for major computing platforms, therefore most Internet-connected computers can run an ACNUC client and access the PBIL databases.

The remote ACNUC access protocol has also been interfaced with two programming languages, C and Python, and the widely used statistical computing environment R [44]. Therefore, it is possible for users to write their own programs in any of these languages in order to access ACNUC databases. Furthermore, the R binding is included into an official R package called seqInR [45]. This package provides various tools for statistical and evolutionary analyses of biological sequences and access to the very large set of libraries available in the R environment.

### FamFetch interface

**Figure 4:**
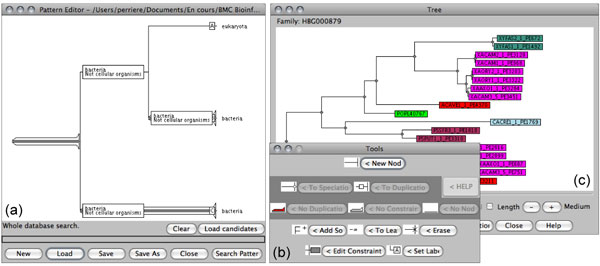
**Three different frames of the FamFetch interface**. Frame (a) is an interactive editor that allows users to build any pattern, node by node and leaf by leaf. Here the pattern entered allows to detect families in which an eukaryotic species is placed within a clade of bacterial species. Frame (b) allows to choose between tools to use in the editor. Tools surrounded by dark grey are those that use the gene duplication predictions, and can be avoided if the user does not want to trust this information. Frame (c) is the tree display. In this frame, sequence are displayed using a colour code corresponding to the taxonomy.

FamFetch is a Java client allowing to access sequence data, as well as the alignments and trees present in HOVERGEN, HOGENOM and HOMOLENS [17]. Starting from the main window of the interface it is possible to access the whole list or a personal subset of families and to make queries to retrieve those matching specific criteria (Figure 4). An equivalent of the Cross Taxa application is also implemented. After selection of a family, the corresponding phylogenetic tree is displayed in the tree window. In this tree, sequences are coloured using a code reflecting the taxonomic position of the corresponding species. A choice of four different editable colouring schemes is proposed to the user. The tree display is active, with options of re-rooting, node swapping, subtree selection or zooming. Clicking on leaves allows users to visualize the entries from UniProt and EMBL or the alignment of the selected sequences.

A major feature of FamFetch is the possibility to retrieve families showing specific tree patterns [18]. The interface integrates a tree pattern editor allowing to define a pattern that will be searched in the set of phylogenetic trees. After the pattern matching operation, the main frame of FamFetch displays the list of matching families. The results can be saved in a file, each pattern being numbered and described with its gene list. Thanks to the possibility to introduce duplications and/or taxonomic data constraints in search patterns, it is possible to easily detect ancient gene duplications or to select orthologous genes. For that purpose, the user only needs to build a pattern in which duplications are forbidden. The whole tree pattern search operation really makes sense with the tree reconciliation performed with RAP. Indeed, with reconciled trees, even hidden paralogies due to duplications followed by gene losses in some lineages are taken into account in the pattern search process.

The use of the tree pattern matching algorithm to retrieve sets of orthologous genes has been previously described [18]. The approach to orthology inference implemented by the RAP tree pattern matching algorithm is very different from that used by most other systems such as COGs [46], OrthoMCL-DB [47] or Inparanoid [48], and is the only one based on phylogenetic analysis. But this tool can be also used for other purposes, and in the case of HOGENOM, it is possible to search for genes that may have been obtained by HGT in some species. HGTs are known to be an important driving force in prokaryotes evolution [31-33], and the question of their detection has raised a lot of methodological problems [49-51]. It is generally admitted that the phylogenetic methods (*i.e.*, the methods based on the use of phylogenetic trees) are the most efficient ones to identify HGTs [50-52]. In order to detect transfers with a database like HOGENOM, the simplest thing to do is to search for anomalous patterns in trees, for instance patterns that are violating the monophyly of a well-established group of species.

**Figure 5:**
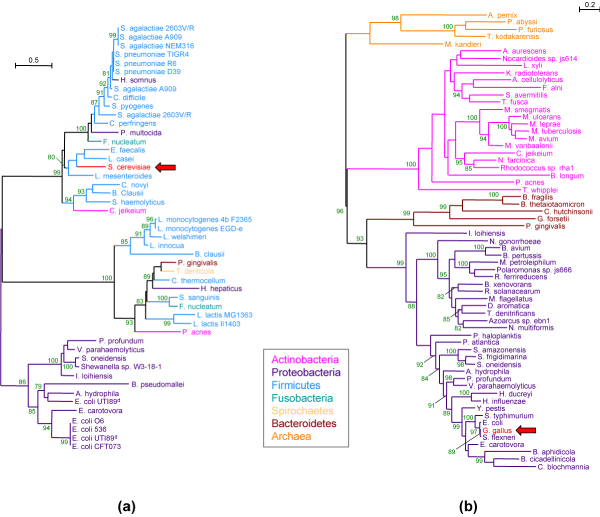
**Exemple of trees containing anomalous patterns involving eukaryotes and bacteria**. A search on the pattern shown in Figure 4 has been performed on HOGENOM release 4, and this search returned a total of 1,304 families. Two trees taken among the 1,304 are shown in this figure. Family HBG082165 (a) corresponds to a conserved hypothetical protein, and it shows a *S. cerevisiae *sequence among Lactobacillales species. Family HBG459980 (b) corresponds to the 3-phosphoshikimate 1-carboxyvinyltransferase enzyme, and it shows a *G. gallus *sequence among Proteobacteria species. Values of the aLRT test are given for the internal branches, and only values with a *P *> 80% are shown.

A possible example of search of this kind is summarized in Figures 4 and 5. In this search, the pattern entered allows to detect families in which an eukaryotic species is placed within a clade of bacterial species (Figure 4). When performed on the release 4 of HOGENOM (February 2008), this search returns 1,304 trees, two of which are shown in Figure 5. Many of these patterns represent probable contaminations rather than real HGTs, an example of this being the presence of *Gallus gallus *among Proteobacteria sequences in HBG459980 family. More plausible is the case of family HBG082165 that shows a possible HGT of a gene encoding an hypothetical protein from a Lactobacillales species to the yeast *Saccharomyces cerevisiae*.

### Programs and data availability

All software, and databases can be freely used and/or downloaded from the PBIL server at . Executable files for Windows, MacOSX, Linux X86 and Solaris of the graphical interface version of Query are distributed, as well as standard C sources for the command-line version. For the FamFetch and RAP programs, Java sources as well as their compiled classes are provided. For the databases, ACNUC index tables, sequence files in EMBL and UniProt format, alignments in Clustal format [53], and trees in Newick format [54] are provided. The seqInR package is available from any Comprehensive R Archive Network (CRAN) mirror. All data used to estimate the reliability of Build_Fam and its comparison with other clustering algorithms can be downloaded at .

## Results and discussion

### Tree reconciliation

The main originality of our system is the possibility to make queries using tree patterns, as this allows users not only to search for orthologs but also for HGTs, gene duplications or any phylogenetic profile of interest. Also, it is possible to perform tree pattern searches on reconciled or non-reconciled databases, the only difference being that duplications need to be described explicitly by the user in a non-reconciled database.

### Clustering algorithm

The comparison of clustering methods revealed that different approaches have different desirable properties. An ideal algorithm for building phylogenetic tree databases would be fast, producing high quality alignments while maximizing species representation in protein families. In terms of speed, Build_Fam indisputably outperformed both TribeMCL and OrthoMCL (respectively less than an hour, 3 hours and 41 hours to cluster 219,951 sequences on a Sun Fire 880, UltraSparc-III, 8 × 900 MHz CPUs, 28 Gbytes RAM). In the clustering procedure, OrthoMCL and TribeMCL always cluster a significantly larger fraction of sequences than Build_Fam with respectively 77–78% and 72–85% against 54–58%, depending on the parameters used for each program (Table 2). As expected, when the Build_Fam similarity threshold is made less stringent, the number of families generated decreases while the average number of sequences per family increases. This average number of sequences per family is usually low because many families have a small number of sequences. An important difference is the fact that Build_Fam and TribeMCL have a tendency to generate a small number of very large families (containing >1,000 sequences), in contrast with OrthoMCL. Overall the clustering criteria appear more stringent in Build_Fam, and therefore the proportion of families that include representatives from more than one kingdom is lower (Table 3). Furthermore when the number of species represented in a family grows, Build_Fam tends to have more sequences per species, and thus to have more redundancy than OrthoMCL (excepted for *I *= 1.1). The tendency of reducing redundancy in families is a build-in characteristic of the OrthoMCL algorithm and is therefore not surprising. It may not, however, be a desirable property for the present databases.

**Table 2:** Clustering results for Build_Fam, OrthoMCL and TribeMCL

	Build_Fam			Ortho_MCL			Tribe_MCL	
Parameters	50/80	40/80	*E*-value	HSP	1.5	4.0	*E*-value	HSP
Nb. clustered seq.	119222	144956	157993	186779	171129	169507	157993	186779
% clustered seq.	54%	66%	72%	85%	78%	77%	72%	85%
Nb. families	20706	17043	19608	19344	23966	31343	19608	19344
Avg. seq./family	5.76	8.51	8.06	9.66	7.14	5.41	8.06	9.66
Families ≥ 1000	1	6	1	1	0	0	1	1
Largest family	1580	2642	1121	1185	479	281	1121	1185
Families sp. = 1	10359 (50%)	8050 (47%)	8379 (43%)	6735 (35%)	7828 (33%)	10134 (32%)	8379 (43%)	6735 (35%)
Families sp. = 50	13 (0.6‰)	34 (2‰)	19 (1‰)	30 (1.6‰)	27 (1.1‰)	5 (0.2‰)	19 (1‰)	30 (1.6‰)
Familles sp. ≥ 25	504 (2.4%)	620 (3.6%)	630 (3.2%)	744 (3.9%)	734 (3.1%)	554 (1.8%)	630 (3.2%)	744 (3.9%)

The parameters used for the algorithms correspond to the similarity/length combination in the case of Build_Fam, to the inflation parameter in the case of OrthoMCL, and to the two scores used in the case of TribeMCL. The three last lines give the number and percentage of families containing only one species (sp. = 1), 50 different species (sp. = 50), and at least 25 different species (sp. ≥ 25).

**Table 3:** Proportion of families integrating sequences from one, two or the three kingdoms of life (Bacteria, Archaea and Eukaryota).

	Build_Fam			OrthoMCL			TribeMCL	
Parameters	50/80	40/80	40/90	1.1	1.5	4.0	*E*-value	HSP
1 kingdom	91%	88%	89%	86%	84%	85%	87%	83%
2 kingdoms	7%	9%	8%	10%	13%	13%	10%	13%
3 kingdoms	2%	3%	3%	4%	4%	2%	3%	4%

Although it detected less universal families, Build_Fam almost consistently produced better alignments than other methods, either for the NorMD index or the number of gaps as detected by Gblocks (Table 4). When the number of sequences or species is low, Build_Fam 50/80 generates alignments that are much better than those obtained with OrthoMCL or TribeMCL. On the other hand, for large and very large families, the quality of the alignments considerably decreases. Considering the largest family generated by Build_Fam 50/80 (1,580 sequences) it happens that it is split by OrthoMCL 1.5 into 104 different families (corresponding to 92% of the total of sequences). The alignments of those 104 families are good as their average NorMD index is >0.5 for 96 families and their average site selection by Gblocks is >60%. There is therefore a tendency of Build_Fam to integrate divergent sequences on very large families.

**Table 4:** Alignment quality results for the Build_Fam, OrthoMCL and TribeMCL algorithms

Algo.	Families	Nb. families	Mean nb. seq.	Mean nb. sp.	Mean %Gbl.	Nb. fam. %Gbl. >50%	Mean NorMD	Nb. fam. NorMD >0.5
BF 50/80		213	10	5.99	**63%**	**172 (80.8%)**	**0.73**	**207 (97.2%)**
BF 40/80		190	10	5.44	51%	96 (50.5%)	0.67	151 (79.5%)
BF 40/90		179	10	6.01	53%	104 (58.1%)	0.65	144 (80.4%)
Ortho 1.1	Seq. = 10	270	10	5.09	36%	76 (28.1%)	0.34	149 (55.2%)
Ortho 1.5		447	10	6.02	38%	136 (30.4%)	0.44	246 (55.0%)
Ortho 4.0		450	10	6.3	45%	186 (41.3%)	0.59	300 (66.7%)
Tribe *E*-value		290	10	5.09	43%	111 (38.3%)	0.59	199 (68.6%)
Tribe HSP		373	10	5.59	31%	77 (20.6%)	0.13	149 (39.9%)

BF 50/80		35	25	16.6	**51%**	**18 (51.4%)**	**0.61**	**26 (74.3%)**
BF 40/80		37	25	16.03	34%	9 (24.3%)	0.46	14 (37.8%)
BF 40/90		45	25	17.27	41%	15 (33.3%)	0.50	25 (55.6%)
Ortho 1.1	Seq. = 25	49	25	15.47	22%	5 (10.2%)	0.05	14 (28.6%)
Ortho 1.5		70	25	16.96	27%	8 (11.4%)	0.33	31 (44.3%)
Ortho 4.0		51	25	18.22	35%	12 (23.5%)	0.45	25 (49.0%)
Tribe *E*-value		38	25	13.683	27%	4 (10.5%)	0.38	11 (28.9%)
Tribe HSP		55	25	14.75	23%	5 (9.1%)	0.13	12 (21.8%)

BF 50/80		7	50	23.29	**35%**	**2 (28.6%)**	**0.49**	2 (28.6%)
BF 40/80		9	50	29.33	28%	0 (0.0%)	0.43	**5 (55.6%)**
BF 40/90		15	50	25.8	22%	1 (6.7%)	0.39	8 (53.3%)
Ortho 1.1	Seq. = 50	23	50	29.91	11%	1 (4.3%)	-0.30	4 (17.4%)
Ortho 1.5		18	50	28.28	17%	1 (5.6%)	0.14	4 (22.2%)
Ortho 4.0		4	50	37	25%	0 (0.0%)	0.48	1 (25.0%)
Tribe *E*-value		11	50	29.64	13%	0 (0.0%)	0.14	0 (0.0%)
Tribe HSP		17	50	30.88	16%	0 (0.0%)	0.00	3 (17.6%)

BF 50/80		107	12.1	10	**60%**	**82 (76.6%)**	**0.66**	**101 (94.4%)**
BF 40/80		102	15.06	10	44%	46 (45.1%)	0.53	63 (61.8%)
BF 40/90		113	13.65	10	48%	51 (45.1%)	0.59	80 (70.8%)
Ortho 1.1	Sp. = 10	121	14.58	10	34%	34 (28.1%)	0.22	59 (48.8%)
Ortho 1.5		224	12.3	10	40%	73 (32.6%)	0.43	119 (53.1%)
Ortho 4.0		221	11.59	10	46%	100 (45.2%)	0.43	142 (64.3%)
Tribe *E*-value		128	14.52	10	47%	56 (43.8%)	0.53	85 (66.4%)
Tribe HSP		172	15.37	10	33%	45 (26.2%)	0.31	74 (43.0%)

BF 50/80		32	30.91	25	**40%**	8 (25.0%)	0.51	19 (59.4%)
BF 40/80		23	42.13	25	28%	7 (30.4%)	0.34	8 (34.8%)
BF 40/90		31	41.74	25	37%	**11 (35.5%)**	0.39	13 (41.9%)
Ortho 1.1	Sp. = 25	36	51	25	16%	2 (5.6%)	0.14	13 (36.1%)
Ortho 1.5		33	37.64	25	28%	5 (15.2%)	0.43	18 (54.5%)
Ortho 4.0		30	27.97	25	35%	7 (23.3%)	**0.52**	**20 (66.7%)**
Tribe *E*-value		26	45.19	25	22%	1 (3.8%)	0.30	10 (38.5%)
Tribe HSP		42	46.29	25	18%	2 4.8%)	0.24	11 (26.2%)

BF 50/80		13	61.38	50	30%	1 (7.7%)	**0.54**	**8 (61.5%)**
BF 40/80		34	181.15	50	23%	4 (11.8%)	0.16	15 (44.1%)
BF 40/90		23	206.3	50	26%	**4 (17.4%)**	0.16	10 (43.5%)
Ortho 1.1	Sp. = 50	55	70.35	50	20%	2 (3.6%)	0.27	18 (32.7%)
Ortho 1.5		27	57.04	50	27%	2 (7.4%)	0.50	14 (51.9%)
Ortho 4.0		5	53.4	50	**32%**	0 (0.0%)	0.51	3 (60.0%)
Tribe *E*-value		19	87.42	50	19%	2 (10.5%)	0.21	5 (26.3%)
Tribe HSP		30	87.67	50	18%	1 (3.3%)	0.16	6 (20.0%)

The different parameters used for Build_Fam (BF), OrthoMCL (Ortho) and TribeMCL (Tribe) are given in the first column. The best scores in four last columns are shown in bold.

On average, the better alignments obtained with Build_Fam for families up to 50 sequences or species can be explained by the double constraint put on the similarity and length of the pair of proteins. This increase in quality is partly counter-balanced by the use of a simple transitive link to incorporate sequences in a family. The use of a complete link would probably ensure an even better alignment quality, but at the cost of many families split. As this phenomenon of families splitting is already important with Build_Fam in its present state, it is probably not worth considering this model of sequence integration for the moment. Remarkably, the 50/80 parameter combination – which was chosen empirically for the first HOBACGEN release [17] – gives better results than the other combinations tested (40/80 and 40/90). This parameter choice thus appears as a good compromise between family size (and therefore family exhaustivity) and alignment quality. As the quality of a phylogenetic tree is the direct consequence of the quality of the corresponding sequence alignment, it is of special importance to have good alignments in our databases. In that context, the lower exhaustivity – materialized by the fact that Build_Fam tends to include only sequences from one kingdom in a family – is acceptable.

### Parallel computing

The sizeable computational volume required by the construction of HOGENOM, HOVERGEN and HOMOLENS has been performed using the computing facilities of the Institut National de Physique Nucléaire et de Physique des Particules (IN2P3). This computing centre provides access to a 2,300 CPU cluster that can efficiently parallelize tasks such as BLAST searches or the construction of thousands of alignments and trees. The use of parallel computing brought a major improvement since computation time has been reduced by a factor of 50 to 100.

## Conclusion

The different databases descibed in this paper are useful tools that has been used in many published biological studies but it might be desirable to create a general gene family database, combining sequence data from all available taxa. One important difficulty is that this would considerably increase the size of many gene families, and hence this would make the phylogenetic trees much more difficult to browse and interpret. Moreover, the global quality of the trees themselves would be drastically lowered because of the difficulty to compute reliable multiple alignments with very large families. Given that users are generally interested only in a particular clade, we decided to maintain three different databases (HOVERGEN, HOGENOM and HOMOLENS), whose content is partly overlapping, but that focus on different clades and different kinds of data (complete genome sequences *vs. *all data available for one clade). Also, we plan to develop a strategy including an incremental all-against-all BLAST search performed on a whole general protein sequence repository collection (such as UniProt). We will provide procedures allowing users to: i) extract a subset from the exhaustive set of protein similarities detected; ii) use this subset to create a specific database. Moreover, we wish to develop tools that would allow the user to automatically edit phylogenetic trees to display only a subset of sequences representative of the taxa of interest.

## Competing interests

The authors declare that they have no competing interests.

## Authors' contributions

SP is in charge of the database maintenance, the development of HOGENOM and HOMOLENS, and the present developments on the Web site. AMA developed the HoSeqI system. JFD developed the RAP program and the tree pattern search algorithm implemented in FamFetch. ASS and VD did the comparisons of the different clustering algorithms. LD conceived the database structure and wrote the Build_Fam program. MG developed the ACNUC system and the Query program, as well as its C API. GP developed the FamFetch interface, the core of the Web interface and wrote the manuscript.
